# Altered white matter microstructure in lupus patients: a diffusion tensor imaging study

**DOI:** 10.1186/s13075-018-1516-0

**Published:** 2018-02-07

**Authors:** Jessika Nystedt, Markus Nilsson, Andreas Jönsen, Petra Nilsson, Anders Bengtsson, Åsa Lilja, Jimmy Lätt, Peter Mannfolk, Pia C Sundgren

**Affiliations:** 1 Department of Clinical Sciences Lund/Diagnostic Radiology, Lund University, Lund, Sweden; 2 Department of Clinical Sciences/Rheumatology, Lund University, Lund, Sweden; 3 Department of Clinical Sciences/Neurology, Lund University, Lund, Sweden; 4 Department of Clinical Sciences/Clinical Imaging and Physiology, Skåne University Hospital, Lund, Sweden; 5 Department of Clinical Sciences/Centre for Imaging and Function, Skåne University Hospital, Lund, Sweden; 6 Department of Neurology, University Hospital of Skåne, Jan Waldenströms gata 15, 205 02 Malmö, Sweden

**Keywords:** Systemic lupus erythematosus, Neuropsychiatric systemic lupus erythematosus, Magnetic resonance imaging, White matter microstructure, Diffusion tensor imaging, Cognitive impairment

## Abstract

**Background:**

The purpose of this study was to investigate whether white matter microstructure is altered in patients suffering from systemic lupus erythematosus (SLE), and if so, whether such alterations differed between patients with and without neuropsychiatric symptoms.

**Methods:**

Structural MRI and diffusion tensor imaging (DTI) were performed in 64 female SLE patients (mean age 36.9 years, range 18.2–52.2 years) and 21 healthy controls (mean age 36.7 years, range 23.3–51.2 years) in conjunction with clinical examination, laboratory tests, cognitive evaluation, and self-assessment questionnaires. The patients were subgrouped according to the American College of Rheumatology Neuropsychiatric Systemic Lupus Erythematosus case definitions into non-neuropsychiatric SLE (nonNPSLE) and neuropsychiatric SLE (NPSLE).

**Results:**

Comparisons between the SLE group and healthy controls showed that the mean fractional anisotropy (FA) was significantly reduced in the right rostral cingulum (*p* = 0.038), the mid-sagittal corpus callosum (CC) (*p* = 0.050), and the forceps minor of the CC (*p* = 0.015). The mean diffusivity (MD) was significantly increased in the left hippocampal cingulum (*p* = 0.017). No significant differences in MD or FA values were identified between NPSLE and nonNPSLE patients. Disease duration among all SLE patients correlated significantly with reduced FA in the CC (*p* < 0.05). No correlations were found between DTI parameters and white matter hyperintensities, SLE Disease Activity Index-2000, Systemic Lupus International Collaborating Clinical/ACR Organ Damage Index, or Montgomery Asberg Depression Rate Score Self-report.

**Conclusions:**

We found alterations of white matter microstructure in SLE patients that were related to disease duration and fatigue. Our results indicate that cerebral involvement in SLE is not isolated to the NPSLE subgroup.

**Electronic supplementary material:**

The online version of this article (10.1186/s13075-018-1516-0) contains supplementary material, which is available to authorized users.

## Background

Systemic lupus erythematosus (SLE) is an autoimmune connective tissue disease that affects many organ systems including the central nervous system [1, 2]. Neuropsychiatric (NP) symptoms are common in SLE [3–5] and are major contributors to increased morbidity and mortality [6, 7]. The distinction between non-neuropsychiatric SLE (nonNPSLE) and neuropsychiatric SLE (NPSLE) is not always clear and accurate diagnosis remains challenging as patients with nonNPSLE as well as HC also might experience neuropsychiatric symptoms, such as headache or cognitive decline [8–10].

The symptoms of NPSLE are diverse and are challenging to diagnose. Symptoms range from mild ones such as headache, fatigue, cognitive decline, and mood disorders, to more severe conditions such as epilepsy, stroke, dementia, and psychosis [3, 8]. The pathogenesis of NPSLE is heterogeneous and largely unknown. Possible mechanisms include autoneuronal antibodies [11, 12], intrathecal production of proinflammatory cytokines that might disintegrate the blood–brain barrier [13], vascular involvement through microangiopathy, chronic diffuse ischemia, thromboembolism, and atherosclerosis [3, 14].

The American College of Rheumatology (ACR) has classified NPSLE as a complex neurologic disorder and set forth 19 case definitions based on the presence of SLE and neuropsychiatric conditions [15]. However, it still remains a challenge to decide whether or not the NP symptoms are due to SLE per se, due to therapy, or simply unrelated to the disease itself since some of the NPSLE manifestations are also common in the general population [4, 8, 16].

Magnetic resonance imaging (MRI) is the standard radiologic imaging modality for NPSLE diagnosis, and commonly demonstrates focal white matter hyperintensities (WMHI) on T2 and FLAIR weighted sequences. However, these alterations can also be seen in nonNPSLE patients as well as in HC [17, 18]. Furthermore, WMHI are not mandatory in NPSLE and up to 45% of NPSLE patients have no visible MRI abnormalities [18–20]. This warrants the development of more exact imaging techniques.

Diffusion tensor imaging (DTI) is a radiological technique that enables imaging of white matter microstructure in vivo, and may detect more subtle changes in tissue microstructure than what can be seen with morphological imaging alone. Due to the structured architecture of axons in the white matter, water diffusion in white matter is anisotropic. By tracking this diffusion with diffusion-weighted MRI and analyzing the data with DTI, the fractional anisotropy (FA) and mean diffusivity (MD) can be computed [21]. FA captures the axon density and the axonal orientation dispersion [22], whereas MD is an indicator of tissue density. High FA and a low MD is an indication of intact myelination and high white matter integrity, whereas reduced FA and increased MD indicates tissue damage, although these interpretations are not without exceptions [23].

DTI allows quantification of FA and MD in specific white matter tracts. It thereby offers a means to assess the microstructure and integrity of intracerebral tracts known to be crucial for cognitive function, which include the corpus callosum (CC), the cinguli bundles, and the unicinatus tracts [24–27]. The CC is the largest bundle of the human brain and connects left and right cerebral hemispheres and is involved in several motor, perceptual, and cognitive functions [28]. The cinguli is part of the limbic system and is involved in attention, memory, and emotions [28]. The uncinate tract connects anterior portions of the temporal lobe with the inferior frontal gyrus and the lower surfaces of the frontal lobe. It is known as an associative bundle thought to belong to the limbic system [28–30].

Previous DTI studies of nonNPSLE and NPSLE patients, although few, indicate diffuse alterations of the white matter microstructure in the CC, the internal anterior capsule, the uncinate tract, and the left cingulum [26, 31, 32]. It seems that these white matter alterations are present already early after SLE diagnosis, are not isolated to NPSLE patients, and might be an underlying mechanism to cognitive impairment in lupus patients [29, 31, 33].

Considering our previous findings from resting state functional MRI (in press), where we demonstrated hypoconnectivity and hyperconnectivity in several crucial resting-state networks in both nonNPSLE and NPSLE patients and that some of these alterations showed a significant correlation to disease duration, we hypothesized that the cerebral microstructure would be altered in NPSLE as well as in nonNPSLE patients and that these alterations would correlate to the disease duration rather than whether they belonged to the NPSLE subgroup or not.

In this study, we aimed to investigate the value of DTI for investigation of alterations in different white matter tracts, and its potential relation to clinical findings, laboratory findings, and, most importantly, to cognitive dysfunction in a well-defined SLE cohort. Our aims were to: investigate DTI abnormalities of cinguli, corpus callosum, and uncinate tracts by analyzing MD and FA; correlate these findings to disease duration, disease activity, fatigue, mood disorders, and cognitive decline in SLE patients; and investigate whether there were significant differences in the WM microstructure between NPSLE and nonNPSLE patients.

## Methods

### Patients and healthy control subjects

In this study, 71 consecutive female patients with SLE with and without NP symptoms (mean age 36.6 years, range 18.2–52.0 years) and 25 healthy, age-matched, female controls (HC) (mean age 38.1 years, range 23.3-52.2 years) were recruited. The local ethical committee approved the study (#2012/4, #2014/748) and informed consent was obtained for all subjects prior to inclusion. Inclusion criteria were female gender, age between 18 and 55 years, and right handedness (as an indication for supposed left hemisphere dominance). Exclusion criteria for healthy controls were male gender, age below 18 or above 55 years, severe mood disorder, autoimmune disease, or any previously diagnosed neurological condition. From the original cohort, seven SLE patients were excluded (one due to previous temporal lobe resection, three due to not being able to fulfill the MRI examination, and another three due to predominant left-handedness) and five HC were excluded (three due to invalid cognitive testing, one due to other autoimmune disease, and one due to dyslexia). Patients were grouped according to the American College of Rheumatology Neuropsychiatric Systemic Lupus Erythematosus (ACR NPSLE) case definitions into SLE with or without neuropsychiatric involvement [15]. Present daily dose and previous glucocorticoid treatment, ongoing and previous treatment with antimalarial medication, DMARDs, biologic therapy, and chemotherapy were registered. After giving their informed consent, all patients underwent rheumatologic and standardized neurologic clinical examination including assessment of disease activity measured with the SLE Disease Activity Index-2000 (SLE-DAI-2 k) [34] and accumulated organ damage measured with the Systemic Lupus International Collaborating Clinics/ACR Organ Damage Index (SDI). All subjects completed the self-assessment questionnaires Montgomery Asberg Depression Rating Scale Self-report (MADRS-S) [35, 36] and Fatigue Severity Score (FSS) [37]. In both the FSS and MADRS-S an elevated score indicates increased levels of fatigue and of depression.

### Ethics, consent, and permissions

The study was approved by the Regional Ethical Review Board in Lund, Sweden (#2012/4, #2014/748) and written informed consent was obtained for all subjects prior to inclusion.

### Neuropsychological evaluation

All subjects underwent a standardized computerized neurocognitive test battery – the Central Nervous System Vital Signs (CNS-VS). Even though the CNS-VS has not been used previously in SLE patients, this test battery was chosen for this study to increase compliance and time effort. However, the CNS-VS has been tested and validated in traumatic brain injury, in dementia, and in patients with attention deficit/hyperactivity disorder (ADHD) [38–40], and used in the evaluation of cognitive function in patients with multiple sclerosis as well as in brain tumor patients [41, 42]. The CNS-VS composite memory, including verbal memory, and visual memory, executive functioning, information processing speed, psychomotor speed, reaction time, complex attention and cognitive flexibility [38, 40]. The test hence captures the cognitive dysfunctions common in SLE patients; such as dysfunction in complex attention, executive skills and psychomotor speed [43, 44]. The CNS VS also measures reaction time/information processing speed, functions found to be of significance as concerns cognitive deficits in SLE patients [43, 44].

### Magnetic resonance imaging

All subjects underwent MRI on a 3 T MR Scanner (Siemens MAGNETOM Skyra, Erlangen, Germany). The imaging protocol included the following conventional sequences; T2W-TSE (33 transverse slices, TE/TR = 100/6870 [ms]), T2W-FLAIR (33 transversal slices, TE/TR/TI = 81/9000/2500 [ms]), and 3D T1W-MPRAGE (1 mm isotropic voxels, TE/TR/TI = 2.54/1900/900 [ms]) and DTI (TR/TE = 7300/73 [ms], voxel size 2×2×2 mm^3^, field of view 256×256×120 mm^3^, *b*-value of 1000 s/mm^2^in 64 directions with 8 additional volumes acquired without diffusion encoding (*b* = 0 s/mm^2^)). The MPRAGE sequence was performed twice; that is, before and after intravenous contrast administration of 0.2 ml/kg of Gadolinium-DOTA (Dotarem^®^; Gothia Medical/Guerbet) in all subjects.

Structural MRI was evaluated visually, blinded and independently, by both an experienced neuroradiologist and a PhD student of neuroradiology. Scans were analyzed for morphological abnormalities, such as acute or old infarcts, hemorrhage, focal or diffuse brain atrophy, focal white matter lesions, pathologic contrast enhancement, and for any additional lesions not related to SLE. WMHI seen on T2/FLAIR images were defined as mild (1–5 discrete WM lesions), moderate (5–10 discrete WM lesions), severe (>10 discrete WM lesions), or very severe (confluent WM lesions).

Prior to DTI analysis, volume registration was performed using ElastiX in order to correct for subject motion and eddy current distortions [22, 45, 46]. DTI maps were calculated using inhouse developed software in Matlab. Tractography was performed with Mrtrix 0.2 [47], using deterministic tractography based on directions obtained using constrained spherical deconvolution (*l*_max_ = 8). A whole-brain tractogram with 10^5^ tracts was generated using an FA threshold of 0.1 as the stop criterion, from which the selected tracts were obtained. Three tracts were chosen for further analysis: the CC, the cinguli, and the uncinate tracts bilaterally. Tracts were identified according to the Catani tractography atlas [28] and segmented semi-automatically. In this process, regions of interests (ROIs) were placed manually in TrackVis (http://www.trackvis.org) in 10 subjects. These ROIs were then warped to the Montreal Neurological Institute (MNI) space, averaged, projected back to the native space of all subjects in the cohort, and used for the tract segmentation. These projection steps used the warp fields produced by nonlinearly registering the FA maps of the subject to the FMRIB58 FA template using FNIRT [48].

**Fig. 1:**
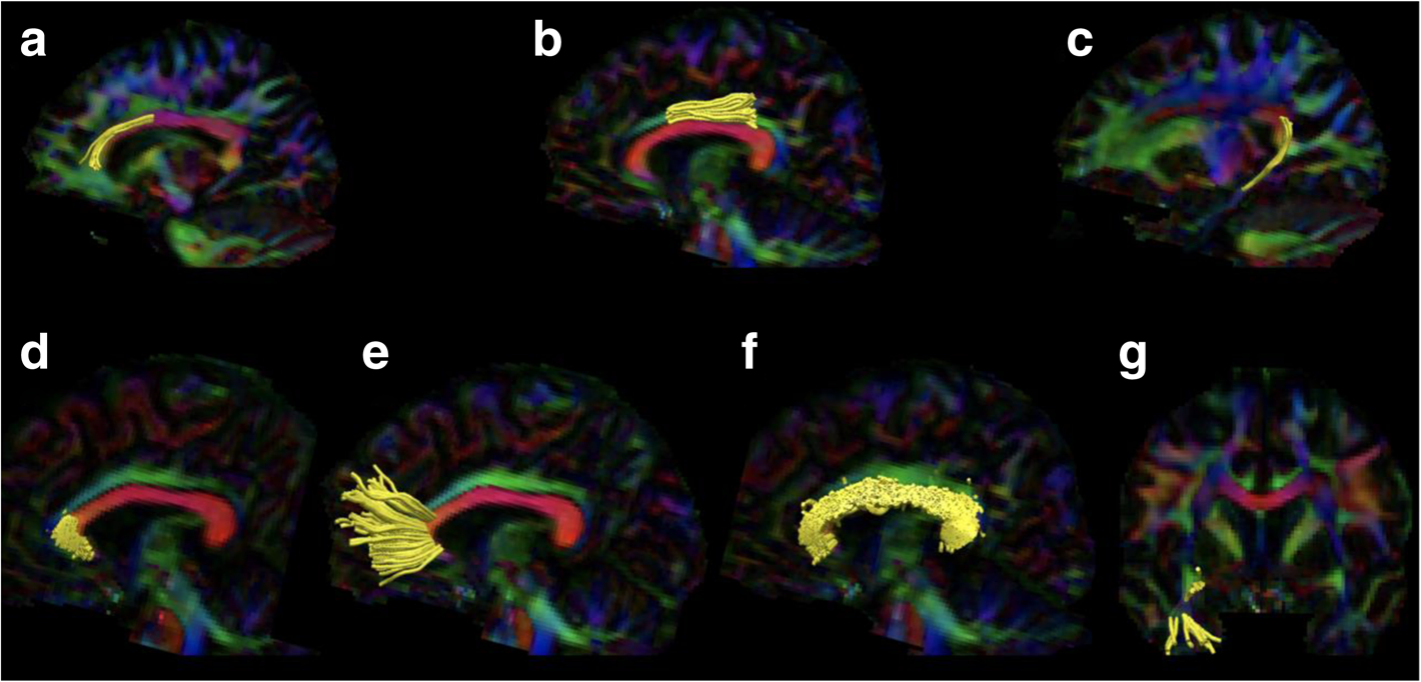
Illustrations of segmented tracts. Upper row illustrates reconstructions of the cingulum with the subgenual cinguli to the left (**a**), the retrosplenal or rostral cingulum in the middle (**b**), and the hippocampal cingulum to the right (**c**). Lower row illustrates the genu of CC (**d**), the forceps minor of the CC (**e**), and the full (genu, body, and splenium) CC (**f**). Lower right corner shows the right uncinate tract (**g**)

All ROIs were controlled visually in each subject and when needed the ROIs were adjusted manually. Parts of the tracts protruding beyond the selecting ROIs were cropped in order to homogenize the selected tracts. An example of the resulting tracts is shown in Fig. 1. Values of FA and MD were then obtained and analyzed for each tract.

### Statistical analysis

Analysis of variance (ANOVA) was used to compare all groups to evaluate for comparisons of differences in DTI metrics. Post-hoc analyses between subgroups were made using the Fisher’s least significant difference test (LSD). Age, FSS index, MADRS-S, cognitive test *z* scores, daily doses of glucocorticoids, and DTI-derived values were evaluated as continuous variables. Disease duration was evaluated both as a continuous variable and a dichotomous one (<2 years; > 10 years). Previous and ongoing pharmacologic treatments were evaluated. Bivariate correlations where tested using Pearson’s correlation coefficient. Nonparametric values were analyzed using the Kruskall–Wallis test. Qualitative variables were analyzed using the chi-square or Mann-Whitney *U* test. Correction for confounding factors was made using ANCOVA. All statistical computations were performed utilizing SPSS (SPSS for Windows, version 24.0; IBM, Armonk, NY, USA). Statistical significance was set at *p* < 0.05, uncorrected.

## Results

### Groups, demographics, and neurocognitive function

The study group, after exclusion, comprised 64 female SLE patients (mean age 36.9 years, range 18.2–52.0 years) and 20 healthy controls (mean age 36.2 years, range 23.3–52.2 years). Age, FSS, and MADRS-S scores, as well as previous and present pharmacologic treatment of all SLE patients, were compared to corresponding values of the HC group. There was no significant difference in age between groups. Compared to HC, SLE patients scored on average significantly higher on FSS (*p* < 10^–4^) and MADRS-S (*p* < 10^–4^). The scores for SLE were lower on average on all domains of the CNS-VS test compared to the HC group, but only psychomotor speed was significantly lower (*p* = 0.001), which indicates some degree of cognitive decline in SLE patients per se (Table 1). After correcting for FSS and MADRS-S scores (i.e., adjusting for the known effect of mood disorders), psychomotor speed (*p* = 0.022) and cognitive flexibility (*p* = 0.034) were significantly lower in the SLE group compared to the HC group.

**Table 1:** Demographics and cognitive test scores (healthy controls/SLE patients)

	Healthy controls (*n* = 20)	SLE patients (all) (*n* = 64)	*p* value
Age	37.3 (33.06–41.46)	36.9 (34.8–39.10)	0.882
MADRS-S	2.85 (1.38–4.32)	12.52 (10.18–14.85)	0.000**
FSS	20.6 (17.0–24.22)	41.0 (37.2–44.83)	0.000**
Cognitive domain (*z* score)			
Memory domain	102.3 (96.8–107.7)	95.42 (91.5–99.4)	0.078
Psychomotor speed	105.7 (100.2–111.2)	95.94 (93.2–98.7)	0.001**
Reaction time	97.5 (88.6–106.4)	89.0 (84.1–93.9)	0.093
Complex attention	106.1(102.5–109.7)	95.75 (89.9–101.6)	0.055
Cognitive flexibility	104.5 (98.8–110.1)	95.2 (89.7–100.7)	0.076

Data presented as mean (95% confidence interval). Characteristics of patients and controls in the cohort after exclusions

SLE systemic lupus erythematosus, MADRS-S Montgomery Asberg Depression Rate Score Self-report, FSS Fatigue Severity Score

**p < 0.01

SLE patients were divided into two groups, nonNPSLE (*n* = 25) and NPSLE (*n* = 39), according to the ACR NPSLE case definitions [12]. Age, disease duration, SLEDAI-2k, SDI, FSS score, MADRS-S score (Table 2), and previous and current pharmacologic treatment (Table 3) were compared between subgroups. There were no significant differences in age, SDI scores, SLEDAI-2k scores, daily dose of glucocorticoids, or disease duration (Table 2). Both groups had lower numerical average scores on all cognitive tests compared to the HC group. The NPSLE group scored significantly lower than the HC group on all domains tested, except on reaction time, while there were no significant differences between nonNPSLE and HC in any of the domains tested. The average FSS and MADRS-S scores of both the nonNPSLE and the NPSLE groups were significantly higher than those of the HC group (*p* < 10^–4^ and *p* < 10^–4^, respectively). The MADRS-S was significantly elevated in the NPSLE group when compared to nonNPSLE (*p* = 0.037). There were significant differences in psychomotor speed (*p* = 0.016), complex attention (*p* = 0.001), and cognitive flexibility (*p* = 0.002) between nonNPSLE and NPSLE after correcting for MADRS-S score (Table 2).

**Table 2:** Demographics and cognitive test scores (HC/nonNPSLE/NPSLE patients)

			*p* value		
	NonNPSLE patients (*n* = 25)	NPSLE patients (*n* = 39)	HC/nonNPSLE	HC/NPSLE	NonNPSLE/NPSLE
Demographics					
MADRS-S	7.8 (4.9–10.7)	15.6 (12.5–18.6)	0.036*	0.000**	0.000**
Duration	11.2 (8.1–14.4)	11.8 (9.1–14.5)			0.783
SDI	0.5 (0.1–0.9)	0.8 (0.4–1.2)			0.312
FSS	38 (32–43)	43 (38–49)	0.000**	0.000**	0.140
SLEDAI-2k	2 (0.9–3.1)	2.7 (1.5–3.8)			0.430
Age	36.0 (32.4–39.7)	37.5 (34.6–40.3)	0.646	0.925	0.524
Daily dose of GCs	4.6 (2.9–6.2)	5.4 (3.7–7.0)			0.510
Cognitive domain					
Memory domain	99 (91–105)	93 (88–98)	0.421	0.033*	0.170
Psychomotor speed	100.1 (96–105)	93 (90–97)	0.090	0.000**	0.016**
Reaction time	92 (85–98)	87(80–95)	0.317	0.063	0.399
Complex attention	106 (101–111)	89 (81–98)	0.997	0.003**	0.001**
Cognitive flexibility	105 (98–112)	89 (82–97)	0.935	0.004**	0.002**

Data presented as mean (95% confidence interval). Characteristics of patients and controls in the cohort after exclusions. Healthy controls (*n* = 20); nonNPSLE patients (*n* = 25); NPSLE patients (*n* = 39)

HC healthy controls, NPSLE neuropsychiatric systemic lupus erythematosus, MADRS-S Montgomery Asberg Depression Rate Score Self-report, SDI Systemic Lupus International Collaborating Clinical/ACR Organ Damage Index, FSS Fatigue Severity Score, SLEDAI-2k SLE Disease Activity Index-2000, GC glucocorticoids

*p < 0.05

**p < 0.01

**Table 3:** Previous and current treatment (nonNPSLE/NPSLE patients)

	NonNPSLE patients (*n* = 25)	NPSLE patients (*n* = 39)	Fischer’s exact test
Previous treatment			
Glucocorticoids	23	34	0.278
Antimalarials	26	36	0.219
DMARDs	20	32	0.249
Current treatment			
Glucocorticoids	19	31	0.487
Antimalarials	21	28	0.207
DMARDs	16	23	0.446
Cyclophosphamide	1	0	0.391
MMF	4	11	0.207
Azathioprin	10	10	0.175
Rituximab	0	1	0.609
IVIG	1	1	0.632
Methotrexate	0	1	0.609
Thalidomide	–	–	–
Leukeran	–	–	–
Belimimab	6	2	0.0034
Antihypertensive treatment	7	13	0.435
SSRI/SNRI	0	2	0.368

There were no significant differences in previous or current treatment strategies between the groups, with the exception of current treatment of belimimab that was more frequent in the nonNPSLE subgroup

NPSLE neuropsychiatric systemic lupus erythematosus, DMARD disease-modifying antirheumatic drug, MMF mycophenolate mofetil, IVIG intravenous immunoglobulins, SSRI/SNRI serotonin reuptake inhibitors/serotonin-norepinephrine reuptake inhibitors

### Magnetic resonance imaging

#### Morphology, WMHI, and brain atrophy

A minimal WMHI lesion load was demonstrated in solely one of the HC (1/20, 5%), and no HC demonstrated moderate or severe load of WMHI. Meanwhile, 42% (27/64) of SLE patients did show mild to severe degrees of WMHI (Table 4).

**Table 4:** Structural MRI and white matter hyperintensities

Groups	No WMHI	Mild WMHI	Moderate WHMI	Severe WMHI	Very severe WMHI
Healthy controls (*n* = 20)	19 (95)	1 (5)	0 (0)	0 (0)	0 (0)
SLE (*n* = 64)	39 (58)	12 (19)	6 (9.4)	7 (11)	2 (3.1)
Subgroups					
NonNPSLE (*n* = 25)	13 (52)	6 (24)	3 (12)	2 (8)	1 (4)
NPSLE (*n* = 39)	15 (54)	6 (39)	3 (8)	5 (13)	1 (2.6)

Data presented as (*N* (%)). Number and percentage of white matter hyperintensities (WMHI) in healthy controls (*n* = 20), all SLE patients (*n* = 64), as well as in subgroups of SLE (nonNPSLE (*n* = 25) and NPSLE (*n* = 39))

SLE systemic lupus erythematosus, NPSLE neuropsychiatric systemic lupus erythematosus

The amount of WMHI were significantly higher in the nonNPSLE group and the NPSLE group compared to the HC group (*p* = 0.015; *p* = 0.023, respectively), while no significant differences were found between nonNPSLE and NPSLE patients (Table 5). There were no significant correlations between the degree of WMHI and *z* scores of cognitive tests. As for the DTI results, there were no significant correlations between MD and WMHI in any of the studied tracts. A negative correlation was found between WMHI and FA in the CC (*r* = –0.230; *p* = 0.027), but not in the other tracts investigated.

**Table 5:** Structural MRI (HC/nonNPSLE/NPSLE patients)

	Mean		*p* value		
	NonNPSLE patients (*n* = 25)	NPSLE patients (*n* = 39)	HC/nonNPSLE	HC/NPSLE	NonNPSLE/NPSLE
Contrast-enhanced lesions	0	0.4	1.0	0.203	0.183
Ischemic lesions	0.04	0.03	0.397	0.535	0.750
Microhemorrhages	0	0.05	1.0	0.219	0.188
Level of atrophy	0.04	0.1	0.640	0.193	0.393
WMHI	0.88	0.77	0.015*	0.023*	0.682
ICH	0	0	-	-	-

Characteristics of patients and controls in the cohort after exclusions. Healthy controls (*n* = 20); nonNPSLE patients (*n* = 25); NPSLE patients (*n* = 39)

MRI magnetic resonance imaging, HC healthy controls, NPSLE neuropsychiatric systemic lupus erythematosus, WMHI white matter hyperintensities, ICH intracerebral hemorrhage

*p < 0.05

**p < 0.01

Small contrast-enhanced lesions were found in 3.1% (2/64) of the SLE patients and micro bleedings were present in another 3.1% (2/64) of the SLE patients. None of the healthy controls demonstrated any pathology or signs of atrophy, whereas 4.7% of the SLE patients showed mild brain atrophy (3/64; one nonNPSLE and two NPSLE patients). One NPSLE patient had moderate cerebellar atrophy (Table 5).

#### FA/MD values in cinguli, uncinate tract, and corpus callosum in the SLE patient cohort

Compared to the HC group, the SLE group exhibited lower FA values in all tracts investigated, although the differences were significant only in the right rostral cingulum (*p* = 0.038), the mid-sagittal CC (*p* = 0.050), and the forceps minor of the CC (*p* = 0.015). MD values were significantly higher in the left hippocampal cingulum (*p* = 0.017). There was also a trend toward higher MD in the forceps minor of the CC (*p* = 0.063) (see Additional file 1: Appendix Table S1 and Table S2).

#### FA/MD values in cinguli, uncinate tract, and corpus callosum when dividing SLE into subgroups: nonNPSLE and NPSLE patients

The nonNPSLE group had significantly lower FA in the right rostral cingulum (*p* = 0.05) compared to the HC group. The MD was higher in the forceps minor of the CC (*p* = 0.027) and in the left hippocampal cingulum (*p* = 0.017). The NPSLE group had significantly lower FA in the midsagittal parts of CC (*p* = 0.032) and forceps minor (*p* = 0.013) and a trend to lower FA in the full CC (*p* = 0.055) compared to the HC group. The MD of the left hippocampal cingulum was significantly higher in the NPSLE group when compared to the HC group (*p* = 0.049). No significant differences in MD or FA were present between nonNPSLE and NPSLE patients (see Additional file 1: Appendix; Table S3 and Table S4).

#### Correlation between white matter integrity and cognitive testing

Scores on cognitive testing of psychomotor speed were positively correlated to higher FA values in the right hippocampal cingulum (*r* = 0.334; *p* = 0.010) in SLE patients. In the NPSLE subgroup, the FA in right subgenual cingulum was significantly correlated to cognitive flexibility (*r* = 0.357; *p* = 0.038), while MD did not correlate to any cognitive test *z* score.

#### FA/MD values in cinguli, uncinate tract, and corpus callosum correlated to fatigue, SDI, SLEDAI-2k, and mood disorders

Neither the MADRS-S, SDI, nor SLEDAI-2k score in SLE patients showed correlation to MD or FA in the investigated tracts. The FSS score was negatively correlated to mean FA in the forceps minor of the CC (*r* = –0.229; *p* = 0.038). The FSS scores in the NPSLE subgroup were correlated to MD in the left hippocampal cingulum (*r* = 0.380; *p* = 0.038). A possible interpretation of these findings could be that decreased microstructural integrity is correlated to increased level of fatigue in SLE patients.

#### FA/MD values and correlation to disease duration

Prolonged disease duration in the SLE group correlated significantly to decreased FA in the forceps minor (*r* = –0.372; *p* = 0.003). This correlation was found also in the NPSLE subgroup (*r* = –0.422; *p* = 0.008). When subdividing all SLE patients into groups on the basis of disease duration, two groups emerged: short-term disease (<2 years) and long-term disease (>10 years). There is no established standard for dividing patients into those with long-term versus short-term disease. Time-frames where chosen to assert potential differences between groups and to enable comparisons with a previous functional resting-state MRI (f-MRI) study [49].

**Fig. 2:**
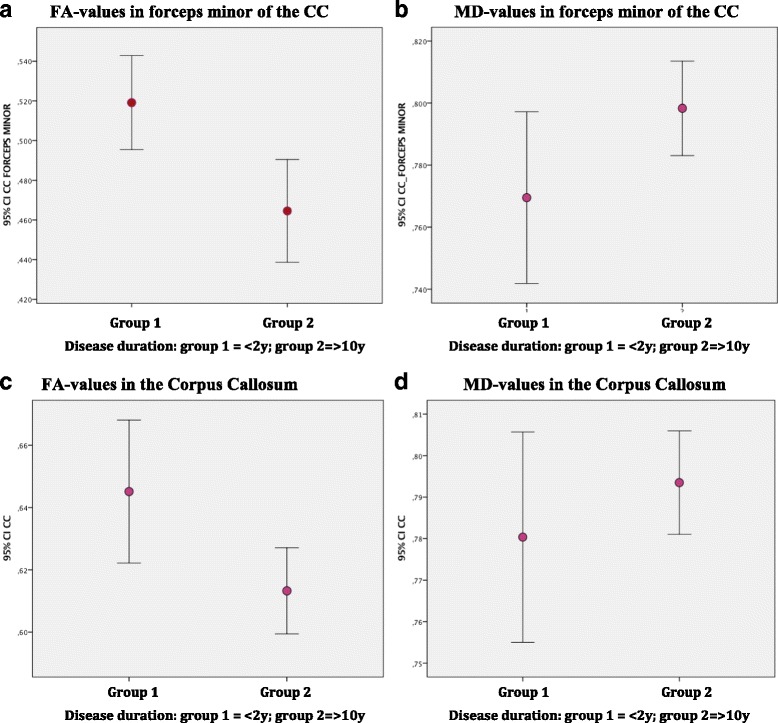
Impact of disease duration on mean diffusivity (MD) and fractional anisotropy (FA) in the corpus callosum (CC) and in the forceps minor of the CC. Mean FA (**a**, **c**) and mean MD (**b**, **d**) in the forceps minor (**a**, **b**) of the CC and the complete CC (**c**, **d**) in SLE patients. Groups stratified based on disease duration: Group 1, < 2 years since diagnosis; Group 2, > 10 years since diagnosis. Significant difference in mean FA values between groups (*p* = 0,014) and a trend in mean MD (*p* = 0.134) in the forceps minor. Same patterns are seen in the full CC with a significant difference in FA (*p* = 0.004) and mean MD (*p* = 0.547) in the CC. CI confidence interval, y years

Compared to the short-term disease group, the long-term disease group showed significantly decreased FA in the mid-sagittal CC (*p* = 0.004), the genu of CC (*p* = 0.008), the forceps minor (*p* = 0.019) (see Fig. 2), and the full CC (*p* = 0.006). This pattern was robust after correcting for age. No significant differences in MD values emerged, but there was a clear trend in the vast majority of studied tracts of increased MD in the long-term group when compared to the short-term group (see Additional file 1: Appendix: Table S5 and Table S6).

## Discussion

In this study, which to our knowledge is the largest study performed with DTI in SLE patients, we investigated abnormalities of white matter tracts hypothesized to be affected in SLE and studied their associations with fatigue, duration of disease, mood disorders, and cognitive function.

One of our main findings was a reduction of FA in all tracts investigated in the SLE group when compared to HC, albeit it was only significantly decreased in the forceps minor of the CC, the right rostral cinguli, and the midsagittal CC. These reductions were not isolated to the NPSLE subgroup, but were also to some degree present in the nonNPSLE group.

There were no significant differences in MD or FA values between the nonNPSLE and the NPSLE groups, further supporting our hypothesis that alterations of the white matter microstructure is part of the SLE disease itself rather than isolated to the patients that clinically manifest neuropsychiatric symptoms and indicating that there is neuronal damage also in SLE patients with no subjective or clinically obvious neuropsychiatric involvement.

These findings are in line with previous studies that found diffuse alterations in white matter microstructure in the CC, uncinate tract, thalamus, and cinguli in NPSLE patients [26, 29, 50]. In nonNPSLE patients with normal-appearing brain on conventional MRI, alterations have been found in the uncinate tract, CC, and left cingulum [26, 29, 31, 51].

Another main finding was that the magnitude of the white matter alterations correlated to disease duration (Fig. 2), suggesting that WM alterations are present already early on in the SLE disease progression and accentuate during the course of the disease. These results are in-line with a previous study suggesting that long-term disease was associated with lower FA and higher diffusivity in the fronto-temporal lobes in NPSLE patients [51, 52]. Similar conclusionshas also been proposed in another study that associated disease duration with alterations in FA and MD values and suggested that disease duration, SDI and SLEDAI-2k scores could serve as possible risk factors for the development of NPSLE [20], and considered disease duration and SDI and SLEDAI-2k scores as possible risk factors for developing NPSLE.

Notably, no association between altered network microstructure and SDI or SLEDAI-2k score was found in or between the SLE subgroups in our study. This is in contradiction to a previous study [32]. However, our cohort had a low mean SLEDAI-2k score, which might explain these diverging results.

Finally, the SLE patients in our cohort performed worse on cognitive testing than the HC. This result was not isolated to the NPSLE subgroup. Differences in cognitive test results between HC and SLE groups were persistent even after correction for FSS and MADRS-S (i.e., adjusted for the known effect of mood disorders on cognitive function). This finding is in accordance with previous studies [53–55].

The pathogenic process responsible for white matter alterations in SLE patients is still unknown, but it can be hypothesized that the vast majority of SLE patients progressively develop neuronal damage and/or demyelination due to the chronic inflammation and that this affect vulnerable brain regions such as the CC, cingulum, and uncinate tracts.

As these processes are probably ongoing in all SLE patients, we believe that brain dysfunction is not isolated to the NPSLE subgroup, but rather a consequence of longstanding inflammation, in contrast to acute severe diffuse or focal NPSLE manifestations. Correlation between white matter alterations and disease duration supports this notion, suggesting that such alterations are present already early on in the SLE disease progression and accentuate during the course of the disease. White matter changes might precede the evolution of fatigue and mood disorders also in lupus patients without clinically obvious neuropsychiatric symptoms. The presence of white matter alterations in both the NPSLE and the nonNPSLE subgroups further supports our hypothesis that white matter alterations are a part of the SLE disease itself rather than isolated to the patients who clinically manifest neuropsychiatric symptoms and indicates that there are neuronal damage also in SLE patients with no subjective or clinically obvious neuropsychiatric involvement.

Our study has four primary limitations. First, although our study is relatively large, the sample size limits the statistical power and no corrections were made for multiple comparisons. Caution is thus required when interpreting single results. However, findings such as diffuse alterations on group level in correlation to disease duration of crucial intracerebral tracts in NPSLE and nonNPSLE patients, or decreased cognitive performance in SLE patients, are in agreement with previous studies [31, 51].

Moreover, the correlations between psychomotor speed and FA in the right hippocampal cingulum in the SLE cohort and between cognitive flexibility and FA in the right subgenual cingulum in the NPSLE subgroup were weak, and it cannot be excluded that the findings were due to the multiple comparisons issue. However, the findings were in line with previous studies indicating that neuronal damage may act as an underlying mechanism to cognitive impairment in SLE patients [33, 51] and may even lead to NPSLE [53, 56].

Second, there was a large variation in previous and actual neuropsychological symptoms between the patients and we did not distinguish active NPSLE patients from patients with inactive NPSLE disease. It is possible that our results would be conclusive if we studied the patients with active NPSLE separately. However, this was beyond the scope of the study. Certainly, the NPACR case definitions may be too permissive in including SLE patients without a clear-cut NP involvement and utilizing a more stringent classification could be of value. However, we have only included patients in the NPSLE group after thorough examination by a neurologist and a rheumatologist, both with special interest in neuropsychiatric lupus and agreement of attribution of NP manifestations to SLE was obtained for each item, which increase the stringency of the present study.

Third, the method we used for cognitive testing (CNS-VS) has not been used previously in SLE patients. However, the CNS-VS has been tested and validated in traumatic brain injury, dementia, and ADHD [40] and also used in evaluation of cognitive function in brain tumor patients [41]. The compliance by the SLE patients and the HC in performing the test was high, and the test was performed with a neuropsychologist present explaining and assisting the subjects prior to the test to optimize the compliance.

Fourth, the pathogenesis of lupus in general and NPSLE in particular is still to a large extent unknown, and confounders such as glucocorticoids, stress, mood disorders, immunosuppressive therapy, and fatigue could influence both the cognitive testing and the imaging findings. It cannot be excluded that our results in part are due to confounding factors.

## Conclusion

Our findings indicate that the white matter microstructure in SLE patients is altered in areas crucial for attention, mood, and cognitive functions. Importantly, this was the case even in patients without NP involvement. Chronic neuropsychiatric symptoms such as fatigue or cognitive impairment may be a consequence of SLE disease activity over time rather than part of an acute NPSLE syndrome more readily identified using the NPACR case definition.

## Additional file

Additional file 1: Appendix Table S1 presenting MD and FA (groups: HC = 0; SLE = 1). Appendix Table S2 presenting MD values in HC (Group 0) and SLE (Group 1). MD values significantly higher in the left hippocampal cingulum in the SLE group when compared to the HC group. Appendix Table S3 presenting FA values (HC/nonNPSLE/NPSLE). Group 0 = HC (*n* = 20), Group 1 = nonNPSLE patients (*n* = 25), Group 2 = NPSLE-patients (*n* = 39). **p* <0.05, ***p* < 0.01. Appendix Table S4 presenting MD values (HC/nonNPSLE/NPSLE). Group 0 = HC (*n* = 20), Group 1 = nonNPSLE patients (*n* = 25), Group 2 = NPSLE patients (*n* = 39). **p* < 0.05, ***p* < 0.01. Appendix Table S5 presenting FA values in relation to disease duration (<2 or > 10 years) in the SLE cohort (*n* = 64). Compared to the short-term disease group, the long-term disease group showed significantly decreased FA in the mid-sagittal CC (*p* = 0.004), the genu of CC (*p* = 0.008), the forceps minor (*p* = 0.019) (see Fig. 2), and the full CC (*p* = 0.006). Appendix Table S6 presenting MD values in relation to disease duration (<2 or > 10 years) in the SLE cohort (*n* = 64). No significant differences in MD values emerged, but there was a clear trend in the vast majority of studied tracts of increased MD in the long-term group when compared to the short time group. **p* < 0.05, ***p* < 0.01. (DOCX 50 kb)

## Abbreviations

ACR: American College of Rheumatology
ACR NPSLE: American College of Rheumatology Neuropsychiatric Systemic Lupus Erythematosus
CNS-VS: Central Nervous System Vital Signs
CC: Corpus callosum
DTI: Diffusion tensor imaging
DMARD: Disease-modifying antirheumatic drug
FSS: Fatigue Severity Score
FA: Fractional anisotropy
rs-fMRI: functional resting-state MRI
HC: Healthy controls
MRI: Magnetic resonance imaging
MD: Mean diffusivity
MADRS: Montgomery Asberg depression rate score
MADRS-S: Montgomery Asberg Depression Rate Score Self-report
NPSLE: Neuropsychiatric systemic lupus erythematosus
nonNPSLE: Non-neuropsychiatric SLE
ROI: Region of interest
SLEDAI-2k: SLE Disease Activity Index-2000
SLE: Systemic lupus erythematosus
SDI: Systemic Lupus International Collaborating Clinical/ACR Organ Damage Index
WM: white matter
WMHI: White matter hyperintensities
