# Supplementary material for: Altered white matter microstructure in lupus patients: a diffusion tensor imaging study
Source: Arthritis Res Ther. 2018 Feb 7;20:21. doi: 10.1186/s13075-018-1516-0 (PMC5803933; doi:10.1186/s13075-018-1516-0)
Supplement: Additional file 1: — Appendix Table S1 presenting MD and FA (groups: HC = 0; SLE = 1). Appendix Table S2 presenting MD values in HC (Group 0) and SLE (Group 1). MD values significantly higher in the left hippocampal cingulum in the SLE group when compared to the HC group. Appendix Table S3 presenting FA values (HC/nonNPSLE/NPSLE). Group 0 = HC (n = 20), Group 1 = nonNPSLE patients (n = 25), Group 2 = NPSLE-patients (n = 39). *p <0.05, **p < 0.01. Appendix Table S4 presenting MD values (HC/nonNPSLE/NPSLE). Group 0 = HC (n = 20), Group 1 = nonNPSLE patients (n = 25), Group 2 = NPSLE patients (n = 39). *p < 0.05, **p < 0.01. Appendix Table S5 presenting FA values in relation to disease duration (<2 or > 10 years) in the SLE cohort (n = 64). Compared to the short-term disease group, the long-term disease group showed significantly decreased FA in the mid-sagittal CC (p = 0.004), the genu of CC (p = 0.008), the forceps minor (p = 0.019) (see Fig. 2), and the full CC (p = 0.006). Appendix Table S6 presenting MD values in relation to disease duration (<2 or > 10 years) in the SLE cohort (n = 64). No significant differences in MD values emerged, but there was a clear trend in the vast majority of studied tracts of increased MD in the long-term group when compared to the short time group. *p < 0.05, **p < 0.01. (DOCX 50 kb) [file 13075_2018_1516_MOESM1_ESM.docx]

**Appendix Table S1: Descriptives MD and FA:** GROUPS: HC=0; SLE=1

| Appendix A: FA |  |  |  |  |  |
| --- | --- | --- | --- | --- | --- |
|  | Groups | Mean | 95% CI for Mean | | P-value |
|  |  |  | Lower Bound | Upper Bound | |
| CING SUBG LEFT | 0 | 0.42 | 0.40 | 0.5 | 0.594 |
|  | 1 | 0.41 | 0.40 | 0.43 |  |
| CING SUBG RIGHT | 0 | 0.37 | 0.35 | 0.39 | 0.39 |
|  | 1 | 0.36 | 0.35 | 0.37 |  |
| CING ROST LEFT | 0 | 0.50 | 0.49 | 0.52 | 0.097 |
|  | 1 | 0.49 | 0.48 | 0.50 |  |
| CING ROST RIGHT | 0 | 0.48 | 0.47 | 0.50 | 0.038* |
|  | 1 | 0.46 | 0.46 | 0.47 |  |
| CING HIPP LEFT | 0 | 0.41 | 0.39 | 0.42 | 0.117 |
|  | 1 | 0.39 | 0.38 | 0.40 |  |
| CING HIPP RIGHT | 0 | 0.38 | 0.36 | 0.40 | 0.53 |
|  | 1 | 0.39 | 0.38 | 0.40 |  |
| UNC LEFT | 0 | 0.33 | 0.31 | 0.36 | 0.106 |
|  | 1 | 0.31 | 0.29 | 0.32 |  |
| UNC RIGHT | 0 | 0.33 | 0.32 | 0.35 | 0.49 |
|  | 1 | 0.32 | 0.31 | 0.34 |  |
| CC FORCEPS MINOR | 0 | 0.52 | 0.51 | 0.54 | 0.015* |
|  | 1 | 0.49 | 0.47 | 0.50 |  |
| CING TOT LEFT | 0 | 1.33 | 1.29 | 1.38 | 0.208 |
|  | 1 | 1.30 | 1.27 | 1.32 |  |
| CING TOT RIGHT | 0 | 1.23 | 1.19 | 1.27 | 0.377 |
|  | 1 | 1.21 | 1.19 | 1.23 |  |
| CC MIDSAG FULL | 0 | 0.67 | 0.66 | 0.68 | 0.05* |
|  | 1 | 0.65 | 0.64 | 0.66 |  |
| CC MIDSAG GENU | 0 | 0.67 | 0.66 | 0.69 | 0.238 |
|  | 1 | 0.66 | 0.65 | 0.67 |  |
| FA CC FULL | 0 | 0.64 | 0.63 | 0.65 | 0.068 |
|  | 1 | 0.62 | 0.62 | 0.63 |  |

**The table illustrates FA-values in: The SLE group (group=1) exhibited lower FA values in all tracts investigated than did HC (group=0) albeit only significantly in the right rostral cingulum (*p* = 0.038), mid-sagittal CC (*p* = 0.050), and the forceps minor of the CC (*p* = 0.015).**

**Appendix Table S2 Descriptives: The table illustrates MD values in HC (group=0) and SLE (group=1). MD values were significantly higher in the left hippocampal cingulum in the SLE-group when compared to the HC-group**

| Appendix B: MD | GROUPS: HC=0; SLE=1 | |  |  |  |
| --- | --- | --- | --- | --- | --- |
|  |  | Mean | 95% Confidence Interval for Mean | | |
|  |  |  | Lower Bound | Upper Bound | Sig. |
| CC MIDSAG FULL | 0 | 0.82 | 0.81 | 0.83 | 0.467 |
|  | 1 | 0.83 | 0.82 | 0.84 |  |
| CC MIDSAG GENU | 0 | 0.79 | 0.77 | 0.81 | 0.786 |
|  | 1 | 0.79 | 0.78 | 0.80 |  |
| CING SUBG LEFT | 0 | 0.73 | 0.71 | 0.75 | 0.763 |
|  | 1 | 0.73 | 0.72 | 0.74 |  |
| CING SUBG RIGHT | 0 | 0.73 | 0.71 | 0.74 | 0.484 |
|  | 1 | 0.73 | 0.72 | 0.74 |  |
| CING ROSTR LEFT | 0 | 0.71 | 0.69 | 0.72 | 0.18 |
|  | 1 | 0.72 | 0.71 | 0.73 |  |
| CING ROSTR RIGHT | 0 | 0.69 | 0.68 | 0.71 | 0.17 |
|  | 1 | 0.71 | 0.70 | 0.72 |  |
| CING HIPP LEFT | 0 | 0.76 | 0.74 | 0.79 | 0.934 |
|  | 1 | 0.76 | 0.75 | 0.78 |  |
| CING HIPP RIGHT | 0 | 0.77 | 0.72 | 0.81 | 0.832 |
|  | 1 | 0,76 | 0.75 | 0.78 |  |
| UNC LEFT | 0 | 0,83 | 0.81 | 0.85 | 0.455 |
|  | 1 | 0,84 | 0.83 | 0.85 |  |
| UNC RIGHT | 0 | 0,80 | 0.79 | 0.82 | 0.793 |
|  | 1 | 0,81 | 0.80 | 0.82 |  |
| MD CC COMP | 0 | 0,79 | 0.78 | 0.80 | 0.551 |
|  | 1 | 0,79 | 0.78 | 0.80 |  |
| CING HIPP LEFT | 0 | 0,72 | 0.70 | 0.74 | 0.017* |
|  | 1 | 0,75 | 0.74 | 0.76 |  |
| UNC LEFT | 0 | 0,89 | 0.86 | 0.92 | 0.091 |
|  | 1 | 0,93 | 0.91 | 0.96 |  |
| UNC RIGHT | 0 | 0,88 | 0.84 | 0.91 | 0.468 |
|  | 1 | 0,90 | 0.87 | 0.92 |  |
| CC FORCEPS MINOR | 0 | 0,77 | 0.75 | 0.79 | 0.063 |
|  | 1 | 0,79 | 0.78 | 0.80 |  |

**Appendix Table S3: FA values (HC/nonNPSLE/NPSLE). Group 0=Healthy controls (n = 20). Group 1 = nonNPSLE-patients (n=25). Group 2=NPSLE-patients (n=39). *P*-value = * <0.05 **<0.01**

| Appendix C: FA | Groups: HCs=0; nonNPSLE=1; NPSLE=2 | | | |  |  |  |
| --- | --- | --- | --- | --- | --- | --- | --- |
|  |  | Mean | 95% Confidence Interval for Mean | | | |  |
|  |  |  | Lower Bound | Upper Bound | *p*-value  (0-1) | *p*-value  (0-2) | *p*-value  (1-2) |
| CING SUBG LEFT | 0 | 0.42 | 0.40 | 0.44 | 0.363 | 0.872 | 0.378 |
|  | 1 | 0.40 | 0.38 | 0.43 |  |  |  |
|  | 2 | 0.42 | 0.40 | 0.44 |  |  |  |
| CING SUBG RIGHT | 0 | 0.37 | 0.35 | 0.39 | 0.601 | 0.345 | 0.683 |
|  | 1 | 0.36 | 0.34 | 0.38 |  |  |  |
|  | 2 | 0.36 | 0.34 | 0.37 |  |  |  |
| CING ROSTR LEFT | 0 | 0.50 | 0.49 | 0.52 | 0.169 | 0.12 | 0.939 |
|  | 1 | 0.49 | 0.48 | 0.50 |  |  |  |
|  | 2 | 0.49 | 0.48 | 0.50 |  |  |  |
| CING ROSTR RIGHT | 0 | 0.48 | 0.47 | 0.50 | 0.05* | 0.074 | 0.716 |
|  | 1 | 0.46 | 0.45 | 0.48 |  |  |  |
|  | 2 | 0.47 | 0.46 | 0.48 |  |  |  |
| CING HIPP LEFT | 0 | 0.41 | 0.39 | 0.42 | 0.194 | 0.143 | 0.954 |
|  | 1 | 0.39 | 0.38 | 0.41 |  |  |  |
|  | 2 | 0.39 | 0.38 | 0.41 |  |  |  |
| CING HIPP RIGHT | 0 | 0.38 | 0.36 | 0.40 | 0.261 | 0.878 | 0.258 |
|  | 1 | 0.40 | 0.38 | 0.41 |  |  |  |
|  | 2 | 0.38 | 0.37 | 0.40 |  |  |  |
| UNC LEFT | 0 | 0.33 | 0.31 | 0.36 | 0.212 | 0.171 | 0.731 |
|  | 1 | 0.31 | 0.28 | 0.33 |  |  |  |
|  | 2 | 0.31 | 0.29 | 0.33 |  |  |  |
| UNC RIGHT | 0 | 0.33 | 0.32 | 0.35 | 0.416 | 0.634 | 0.66 |
|  | 1 | 0.32 | 0.30 | 0.35 |  |  |  |
|  | 2 | 0.33 | 0.31 | 0.34 |  |  |  |
| CC FORCEPS MINOR | 0 | 0.52 | 0.51 | 0.54 | 0.081 | 0.013* | 0.509 |
|  | 1 | 0.49 | 0.47 | 0.51 |  |  |  |
|  | 2 | 0.48 | 0.46 | 0.50 |  |  |  |
| CING TOT LEFT | 0 | 1.33 | 1.29 | 1.38 | 0.217 | 0.298 | 0.75 |
|  | 1 | 1.29 | 1.26 | 1.33 |  |  |  |
|  | 2 | 1.30 | 1.26 | 1.34 |  |  |  |
| CING TOT RIGHT | 0 | 1.23 | 1.19 | 1.27 | 0.634 | 0.312 | 0.599 |
|  | 1 | 1.22 | 1.18 | 1.26 |  |  |  |
|  | 2 | 1.21 | 1.19 | 1.23 |  |  |  |
| CC MIDSAG FULL | 0 | 0.67 | 0.66 | 0.68 | 0.222 | 0.032* | 0.368 |
|  | 1 | 0.66 | 0.64 | 0.67 |  |  |  |
|  | 2 | 0.65 | 0.64 | 0.66 |  |  |  |
| CC MIDSAG GENU | 0 | 0.68 | 0.66 | 0.69 | 0.522 | 0.176 | 0.476 |
|  | 1 | 0.67 | 0.65 | 0.68 |  |  |  |
|  | 2 | 0.66 | 0.64 | 0.67 |  |  |  |
| FA CC FULL | 0 | 0.64 | 0.63 | 0.65 | 0.22 | 0.055 | 0.516 |
|  | 1 | 0.63 | 0.61 | 0.64 |  |  |  |
|  | 2 | 0.62 | 0.61 | 0.63 |  |  |  |

**Appendix Table S4: MD values (HC/nonNPSLE/NPSLE). Group 0=Healthy controls (n = 20). Group 1 = nonNPSLE-patients (n=25). Group 2=NPSLE-patients (n=39). *P*-value = * <0.05 **<0.01**

| Appendix D: MD |  | Mean | 95% CI for the Mean | | p-value  (0-1) | p-value  (0-2) | p-value  (1-2) |
| --- | --- | --- | --- | --- | --- | --- | --- |
|  |  |  | Lower Bound | Upper Bound | |  |  |
| CC MIDSAG FULL | 0 | 0.82 | 0.81 | 0.83 | 0.762 | 0.369 | 0.54 |
|  | 1 | 0.83 | 0.81 | 0.84 |  |  |  |
|  | 2 | 0.83 | 0.82 | 0.85 |  |  |  |
| CC MIDSAG GENU | 0 | 0.79 | 0.77 | 0.81 | 0.974 | 0.661 | 0.611 |
|  | 1 | 0.79 | 0.78 | 0.80 |  |  |  |
|  | 2 | 0.79 | 0.77 | 0.80 |  |  |  |
| CING SUBG LEFT | 0 | 0.73 | 0.71 | 0.75 | 0.601 | 0.391 | 0.601 |
|  | 1 | 0.74 | 0.73 | 0.75 |  |  |  |
|  | 2 | 0.73 | 0.71 | 0.74 |  |  |  |
| CING SUBG RIGHT | 0 | 0.73 | 0.71 | 0.74 | 0.581 | 0.509 | 0.984 |
|  | 1 | 0.73 | 0.71 | 0.75 |  |  |  |
|  | 2 | 0.73 | 0.72 | 0.74 |  |  |  |
| CING ROSTR LEFT | 0 | 0.71 | 0.69 | 0.72 | 0.231 | 0.23 | 0.913 |
|  | 1 | 0.72 | 0.71 | 0.73 |  |  |  |
|  | 2 | 0.72 | 0.71 | 0.73 |  |  |  |
| CING ROSTR RIGHT | 0 | 0.69 | 0.68 | 0.71 | 0.3 | 0.73 | 0.795 |
|  | 1 | 0.70 | 0.69 | 0.72 |  |  |  |
|  | 2 | 0.71 | 0.69 | 0.72 |  |  |  |
| CING HIPP LEFT | 0 | 0.72 | 0.70 | 0.74 | 0.017* | 0.049* | 0.5 |
|  | 1 | 0.75 | 0.73 | 0.77 |  |  |  |
|  | 2 | 0.75 | 0.73 | 0.76 |  |  |  |
| CING HIPP RIGHT | 0 | 0.77 | 0.72 | 0.81 | 0.321 | 0.704 | 0.117 |
|  | 1 | 0.75 | 0.73 | 0.77 |  |  |  |
|  | 2 | 0.77 | 0.76 | 0.79 |  |  |  |
| UNC LEFT | 0 | 0.83 | 0.81 | 0.85 | 0.365 | 0.618 | 0.601 |
|  | 1 | 0.84 | 0.82 | 0.86 |  |  |  |
|  | 2 | 0.84 | 0.82 | 0.85 |  |  |  |
| UNC RIGHT | 0 | 0.80 | 0.79 | 0.82 | 0.901 | 0.755 | 0.849 |
|  | 1 | 0.81 | 0.79 | 0.82 |  |  |  |
|  | 2 | 0.81 | 0.80 | 0.82 |  |  |  |
| MD CC FULL | 0 | 0.79 | 0.78 | 0.80 | 0.474 | 0.687 | 0.688 |
|  | 1 | 0.79 | 0.78 | 0.81 |  |  |  |
|  | 2 | 0.79 | 0.78 | 0.80 |  |  |  |
| CC FORCEPS MINOR | 0 | 0.77 | 0.75 | 0.79 | 0.027* | 0.223 | 0.97 |
|  | 1 | 0.80 | 0.78 | 0.82 |  |  |  |
|  | 2 | 0.79 | 0.77 | 0.80 |  |  |  |

| Appendix E: FA | DURATION (<2y or >10y) | |  |  |  |
| --- | --- | --- | --- | --- | --- |
|  |  | Mean | 95% Confidence Interval for Mean | | |
|  |  |  | Lower Bound | Upper Bound | Sign. (p-value) |
| CC MIDSAG FULL | <2 | 0.68 | 0.66 | 0.69 | 0.04* |
|  | >10 | 0.64 | 0.63 | 0.66 |  |
| CC MIDSAG GENU | <2 | 0.70 | 0.68 | 0.72 | 0.008** |
|  | >10 | 0.65 | 0.63 | 0.67 |  |
| CC FULL | <2 | 0.65 | 0.64 | 0.67 | 0.006** |
|  | >10 | 0.61 | 0.60 | 0.63 |  |
| CING SUBG LEFT | <2 | 0.48 | 0.40 | 0.56 | 0.785 |
|  | >10 | 0.47 | 0.44 | 0.50 |  |
| CING SUBG RIGHT | <2 | 0.42 | 0.37 | 0.48 | 0.385 |
|  | >10 | 0.39 | 0.36 | 0.42 |  |
| CING ROSTR LEFT | <2 | 0.59 | 0.56 | 0.62 | 0.195 |
|  | >10 | 0.56 | 0.55 | 0.58 |  |
| CING ROSTR RIGHT | <2 | 0.54 | 0.49 | 0.59 | 0.941 |
|  | >10 | 0.54 | 0.52 | 0.55 |  |
| CING HIPP LEFT | <2 | 0.37 | 0.32 | 0.42 | 0.339 |
|  | >10 | 0.39 | 0.37 | 0.41 |  |
| CING HIPP RIGHT | <2 | 0.39 | 0.35 | 0.43 | 0.874 |
|  | >10 | 0.39 | 0.37 | 0.42 |  |
| UNC LEFT | <2 | 0.37 | 0.36 | 0.38 | 0.149 |
|  | >10 | 0.35 | 0.34 | 0.36 |  |
| UNC RIGHT | <2 | 0.39 | 0.36 | 0.42 | 0.249 |
|  | >10 | 0.37 | 0.36 | 0.38 |  |
| CC FORCEPS MINOR | <2 | 0.52 | 0.50 | 0.55 | 0.019* |
|  | >10 | 0.47 | 0.44 | 0.49 |  |
| CING TOT LEFT | <2 | 1.27 | 1.16 | 1.37 | 0.661 |
|  | >10 | 1.28 | 1.24 | 1.32 |  |
| CING TOT RIGHT | <2 | 1.21 | 1.14 | 1.29 | 0.883 |
|  | >10 | 1.21 | 1.17 | 1.24 |  |

**Appendix Table S5: FA values in relation to disease duration (<2 or >10 y) in the SLE cohort (n=64). Compared to the short-term disease group, the long-term disease group showed significantly decreased FA in the mid-sagittal CC (*p =* 0.004), the genu of CC (*p* = 0.008), forceps minor (*p* = 0.019), (see fig 2) and the full CC (*p* = 0.006).**

***p*-value = * <0.05 **<0.01**

| Appendix F: MD | DURATION (<2y or >10y) |  |  |  | |  | |
| --- | --- | --- | --- | --- | --- | --- | --- |
|  |  | Mean | 95% CI for Mean | | | | p-value |
|  |  |  | Lower Bound | | Upper Bound | |  |
| CC MIDSAG FULL | <2 | 0.82 | 0.78 | | 0.86 | | 0.507 |
|  | >10 | 0.83 | 0.82 | | 0.85 | |  |
| CC MIDSAG GENU | <2 | 0.77 | 0.74 | | 0.80 | | 0.169 |
|  | >10 | 0.79 | 0.78 | | 0.80 | |  |
| CING SUBG LEFT | <2 | 0.73 | 0.69 | | 0.76 | | 0.728 |
|  | >10 | 0.73 | 0.72 | | 0.74 | |  |
| CING SUBG RIGHT | <2 | 0.71 | 0.68 | | 0.75 | | 0.123 |
|  | >10 | 0.74 | 0.73 | | 0.75 | |  |
| CING ROSTR LEFT | <2 | 0.70 | 0.67 | | 0.74 | | 0.215 |
|  | >10 | 0.72 | 0.71 | | 0.73 | |  |
| CING ROSTR RIGHT | <2 | 0.70 | 0.66 | | 0.73 | | 0.434 |
|  | >10 | 0.71 | 0.70 | | 0.72 | |  |
| CING HIPP LEFT | <2 | 0.76 | 0.73 | | 0.80 | | 0.169 |
|  | >10 | 0.76 | 0.74 | | 0.78 | |  |
| CING HIPP RIGHT | <2 | 0.76 | 0.67 | | 0.85 | | 0.803 |
|  | >10 | 0.75 | 0.74 | | 0.77 | |  |
| UNC LEFT | <2 | 0.82 | 0.79 | | 0.85 | | 0.251 |
|  | >10 | 0.84 | 0.83 | | 0.86 | |  |
| UNC RIGHT | <2 | 0.79 | 0.76 | | 0.82 | | 0.2 |
|  | >10 | 0.81 | 0.79 | | 0.82 | |  |
| CC FULL | <2 | 0.78 | 0.75 | | 0.81 | | 0.259 |
|  | >10 | 0.79 | 0.78 | | 0.81 | |  |
| CC FORCEPS MINOR | <2 | 0.77 | 0.74 | | 0.80 | | 0.078 |
|  | >10 | 0.80 | 0.78 | | 0.81 | |  |

**Appendix Table S6: MD values in relation to disease duration (<2 or >10 y) in the SLE cohort (n=64). No significant differences in MD values emerged, however there was a clear trend in the vast majority of studied tracts of increased MD in the long-term group when compared to the short time group. *P*-value = * <0.05 **<0.01**
